# Comprehensive transcriptional profiling of prion infection in mouse models reveals networks of responsive genes

**DOI:** 10.1186/1471-2164-9-114

**Published:** 2008-03-03

**Authors:** Garrett Sorensen, Sarah Medina, Debra Parchaliuk, Clark Phillipson, Catherine Robertson, Stephanie A Booth

**Affiliations:** 1 Prion Diseases Program, National Microbiology Laboratory, Public Health Agency of Canada, Winnipeg, MB, R3E 3R2, Canada; 2 Department of Medical Microbiology, University of Manitoba, Winnipeg, MB, R3E 0W3, Canada

## Abstract

**Background:**

Prion infection results in progressive neurodegeneration of the central nervous system invariably resulting in death. The pathological effects of prion diseases in the brain are morphologically well defined, such as gliosis, vacuolation, and the accumulation of disease-specific protease-resistant prion protein (PrP^Sc^). However, the underlying molecular events that lead to the death of neurons are poorly characterised.

**Results:**

In this study cDNA microarrays were used to profile gene expression changes in the brains of two different strains of mice infected with three strains of mouse-adapted scrapie. Extensive data was collected and analyzed, from which we identified a core group of 349 prion-related genes (PRGs) that consistently showed altered expression in mouse models. Gene ontology analysis assigned many of the up-regulated genes to functional groups associated with one of the primary neuropathological features of prion diseases, astrocytosis and gliosis; protein synthesis, inflammation, cell proliferation and lipid metabolism. Using a computational tool, Ingenuity Pathway Analysis (IPA), we were able to build networks of interacting genes from the PRG list. The regulatory cytokine TGFB1, involved in modulating the inflammatory response, was identified as the outstanding interaction partner for many of the PRGs. The majority of genes expressed in neurons were down-regulated; a number of these were involved in regulatory pathways including synapse function, calcium signalling, long-term potentiation and ERK/MAPK signalling. Two down-regulated genes coding for the transcription regulators, EGR1 and CREB1, were also identified as central to interacting networks of genes; these factors are often used as markers of neuronal activity and their deregulation could be key to loss of neuronal function.

**Conclusion:**

These data provides a comprehensive list of genes that are consistently differentially expressed in multiple scrapie infected mouse models. Building networks of interactions between these genes provides a means to understand the complex interplay in the brain during neurodegeneration. Resolving the key regulatory and signaling events that underlie prion pathogenesis will provide targets for the design of novel therapies and the elucidation of biomarkers.

## Background

Prion diseases or transmissible spongiform encephalopathies (TSEs) have long incubation times and are characterized by progressive neurodegeneration leading to death [1]. Brain tissue adopts a spongy appearance and a modified form of a normal host protein, the prion protein, is deposited. It is this conformational isomer of prion protein (PrP^Sc^) that is believed to be the infectious agent. The progressive neurodegeneration resulting from infection with prion agents involves diverse cell types, a variety of cellular interactions, and multiple genes. Genomic and proteomic techniques can be used to measure the relative abundance of messenger RNA transcripts and proteins in cells and tissues. The differential expression of these molecules between normal and diseased tissues provides information that can be used to determine the precise molecular mechanisms involved in neurodegeneration in prion disease.

A number of studies to determine the gene expression changes that accompany prion diseases have been performed in different laboratories, including our own [2-9]. Hundreds of differentially expressed genes in various models of TSEs have been identified; many repeatedly. In the present study we have performed an extensive global analysis of gene expression in two strains of mice infected with three strains of scrapie, to create a list of genes consistently deregulated in multiple scrapie models. This gene list includes many previously described, as well as a number of novel genes. Comprehensive bioinformatics analysis of these data has allowed us to make substantial progress in defining networks of interacting genes which operate during neurodegeneration in mouse scrapie.

## Results and discussion

### Identification of consistently deregulated genes in mouse prion disease

C57BL/6 mice were inoculated by intracerebral infection of brain homogenate from mice clinically infected with the ME7, 79a and 22A strains of scrapie. In addition VM mice were also inoculated with the 22A scrapie strain. Mice were sacrificed at the onset of clinical diseases as manifested by uncoordinated gait, flaccid paralysis of the hind limbs, rigidity and abolishment of the righting reflex. Brain tissue was collected from these mice and the RNA isolated. Mouse CNS gene expression was analysed by two-colour microarray experiments using an in house manufactured 11 K mouse cDNA microarray [2]. RNA from individual infected mice was hybridized to each array versus pooled reference RNA from an equivalent number of age-matched, mock-infected control mice. In total we hybridized 34 different samples to microarrays in this experiment; 8–10 individual mice from each of the four sample groups were individually processed for separate microarrays. Hierarchical clustering shows that the patterns of gene expression are for the most part common to the different mouse models (Additional file 1). We used the program EDGE to identify genes that were differentially expressed in mouse brain during clinical disease [9]. We used a P value cut-off of 0.05 as the criteria for selection of significantly differentially expressed genes.

In our previous study we found very few genes to be significantly differentially expressed during pre-clinical disease and so in this study we limited analysis to those genes that were up- or down-regulated at the late clinical stage of the disease [2]. The resulting gene lists contained between 917 and 1076 significant genes from each sample group tested; a sample group contained 8–10 individual infected mice and equal numbers of age matched, mock-infected control mice. These lists of significant genes were used to compile a master list of genes based on the criteria that significant differential expression was identified in at least three of the experimental sample groups tested. The 349 genes that selected were termed "prion-related genes" (PRGs); a complete list of these differentially expressed genes including annotation is provided as Additional File 2. This list of genes includes many that have been previously identified as deregulated in neurodegenerative diseases, these genes are marked on the table; a number of novel genes were also identified. We confirmed deregulation of ~50% of the 349 PRG's (marked in bold in Additional File 2) to be accordingly deregulated in a fourth strain of scrapie, RML, using whole genome mouse arrays purchased from Agilent.

We used an online tool based on a curated database, the Ingenuity Pathways Knowledge Base (IPKB), to annotate genes and to determine potential regulatory networks and pathways. The IPKB contains information on human, mouse, and rat genes including annotations, synonyms and over 1.4 million published biological interactions between genes, proteins and drugs. This database is continually updated and supplemented with curated relationships taken from MEDLINE abstracts; i.e. each gene interaction held in the IPKB is supported by published information. Thus, the IPKB thus provides a framework by which lists of genes identified by large-scale microarray studies can be annotated in terms of their functional relationships, and those that have been shown to interact.

We first identified key biological functions and/or diseases that contain a disproportionately high number of genes from the PRG list in relation to the assayed gene population as a whole; the assayed gene group is the total genelist from the microarray used. Representative categories of biological functions and/or diseases in which PRG genes are over-represented (P values < 0.001) are provided in Table 1. This list of genes includes members of gene families that have been identified in previous studies including genes involved in; lysosome organization and biogenesis, immune cell activation and inflammatory response, lipid metabolism, apoptosis, protein biosynthesis and proteolysis, nervous system function and synaptic transmission, and cytoskeleton organization and biogenesis.

**Table 1:** Summary of the highest represented functional groups of genes from the PRG list. Up-regulated genes are in bold type, down-regulated genes in italics.

**Top Biological Functions**	**Genes**
Lipid Metabolism	*PRDX6*, **HEXB**, **APOD**, *OSBP*, **BMPR1B**, **PTPMT1**, *LTC4S*, *DHCR24*, **APOE**, **SGPP1**, *PITPNC1*, *HMGCS2*, **OSBPL1A**, **GRN**, *CYP46A1*, **ABCA1**
Nervous system function and synaptic transmission	**MBP**, **PMP22**, **PLP1**, *CBLN1*, **APOE**, *CHRNB4*, *YWHAG*, *CART*, *MAL*, *VAMP1*, *VAMP2*, *SYT2*, *SYN2*, *SNPH*, *RPH3A*, *CAMK2A*, *SEPT*, **CTNNB1**, *DNER*, *THY1*, **CD9**, **RTN4**, *NRXN1*, *NRXN2*, **PTN**
Cytoskeleton organization and biogenesis	**SGCB**, **APOE**, **TMSB4X**, *THY1*, **CAPZB**, **RHOF**, **LASP1**, **CNN3**, *ARPC3*, *YWHAG*, *TLN1*, *TUBB2A*, **ERBB2IP**, **DCTN2**, **MYL6**, **GFAP**, **MAP1LC3B**, *ALS2CR2*, *NEFL*, *MYH11*, **EPB49**
Protein biosynthesis and folding	**RPL11**, **RPS3**, **RPS27**, **FAU**, **RPS9**, **WBSCR1**, **SECISBP2**, **RPL9**, **EEF1A1**, **RPL24**, **RPL26**, **CCT6A**, **ERP29**, *HSPB3*, *PPIL2*, **CRYAB**, *DNAJA1*, *WBSCR18*, **HSPA9B**, *HSP90AB1*
Lysosome, organization and biogenesis	**LAPTM5**, **CTSS**, *PRDX6*, **MCOLN1**, **PPT1**, **CTSB**, **LAMP2**, **HEXB**, **CTSK**, **CTSH**
Proteolysis	**UCHL5**, **SNX17**, **PSMA3**, **CTSS**, **SHFM1**, **ASRGL1**, **CTSB**, **PGCP**, **CTSK**, **CTSH**, *MMP14*
Apoptosis	**APOE**, **CTSB**, **LY86**, **SGK**, **HIPK2**, *MAL*, *NFKB1A*, **PPM1F**, **APLP1**, **TRAF5**, **CLU**, **CTNNB1**, *PRKCE*, **TIMM50**, *ALS2CR2*, **SOX9**, **DDIT3**, **TM2D1**, **SGPP1**, *THY1*, **TNF**, **SPP1**, *YWHAG*, **RTN4**, **TGFB1**, **HSPA9B**
Immune cell activation and inflammatory response	*EGR1, THY1*, **TGFB1**, **SPP1**, **IGBP1**, **PRKCD**, **CBLB**, **LY86**

### Identification of biologically relevant networks

To further investigate the global expression response to infection with the prion agent, and to define how individual regulated genes interact to have a coordinated role in specific pathways, we identified potential networks of interacting PRG's using the Ingenuity Pathway Analysis tool. The methodology integrates genomic data with mining techniques to predict protein networks that comprise protein-protein interactions and other functional linkages. Each potential network is given a score, which is a probabilistic fit between the networks and a list of biological functions stored in the IPKB. The score takes into account the number of Focus genes (from the PRG list) in the network, and the size of the network, to approximate how relevant it is to the original list of genes; these scores are used to rank the networks. As the list was extensive we restricted our analysis to those networks that had scores >9 (a score of 3 or greater was considered significant (*p *< 0.001). We identified 22 networks of potentially interacting genes from amongst the PRGs; the top 5 networks are provided in Table 2.

**Table 2:** List of Ingenuity networks generated by mapping the focus genes (PRGs) that were differentially expressed during neurodegeneration.

	**Network genes (focus genes in bold)**	**Score**^#^	**Focus Genes**	**Top Functions**
1	**ALPL**, **CALM2**, **CLU**, **CNN3**, **CTSK**, **EEF1A1**, **EGR1**, **G3BP**, **GFRA2**, **GORASP2**, **GTF2I**, **HSPA9B(includesEG:3313)**, **KLF16**, **KRT1**, **TC4S**, **MMP14**, **MYH11**, **MYL6**, **P4HA1**, **PDGFC**, **PGCP**, **PGRMC1**, **PRKCD**, **PRKCG**, **PSMA3**, **RAB1A**, **RAP1A**, **RET**, **SGPP1**, **SLC7A1**, **SMAD5**, **TBCD**, **TGFB1**, **TUBB2A**, **WBSCR1**	52	35	Tissue Development, Cell Death, Endocrine System
2	**ACTB**, **APOD**, **BTF3**, **CAPZB**, **CASK**, **CASKIN1**, **CCT6A**, **CREB1**, **CTNNB1**, **DBI**, **EPB41**, **ERBB2IP**, **ERP29**, **FGFR3**, **GJA1**, **GTF2F1**, **GTF2H1**, **HSP90AB1**, **ID4**, **LRRFIP2(includesEG:9209)**, **NF2**, **NRXN1**, **PKD1**, **PRKCE**, **PTN**, **PTPRZ1**, **SDC3**, **SFRS1**, **SGK**, **SLC9A3R1**, **SST**, **TF**, **TNRC6A**, **TOB2**, **TRPC4**	52	35	Behavior, Cellular Movement, Cell Cycle
3	**APOE**, **B2M**, **CEBPD**, **CTSB**, **CTSS**, **HEXB**, HLA-DMA, HLA-F, **HMGCS2**, **HRSP12**, IFNG, IL6, IL17A, IL1RL1, ITGB3, **LAPTM4A**, **LY86**, MCHR1, **MLYCD**, **NR1D1**, NR1H2, **OSBPL1A**, PLA2G5, POLR2D, **POLR2F**, **POLR2G**, POLR2I, POLR2J, POLR2K, **POMP (includesEG:51371)**, RXRA, **SLC37A4**, TAP1, **TLN1**, **ZMYND**	19	19	Inflammatory Disease, Connective Tissue Disorders, Infectious Disease
4	**ACTB**, ALB, ATOX1, BID, CASP4, **CEBPD**, **CLCN2**, **CLU**, **CST3**, **CTSB**, **CTSH**, **CTSS**, **DCTN2**, **DDIT3**, DEFB103A, DEFB4(includesEG:1673), FKBP4, GFAP, GH1, **GLUD1**, **GTPBP4**, IRF1, MLLT4, **NCKIPSD**, **NRXN2**, SLPI, **SNPH**, TANK, **TINF2**, TP53, XRCC5, XRCC6, YWHAA, **YWHAB**, **YWHAG**	17	18	Cancer, Cell Death, Tumor Morphology
5	A2M, ALB, APBB3, **APLP1(includesEG:333)**, APOA1, **APOE**, APP, **ARPC3**, **CEBPD**, **CLU**, **CREB1**, **CST3**, CTSD, DAB1, **DOCK3**, **DPYSL2**, FOXA1, FOXA2, FOXA3, **HIP2**, HMOX1, HSPG2 (includesEG:3339), LIF, MAPT, ONECUT1, PAX3, PTGS2, **RAB11B**, **RASA2**, **RLBP1**, **SPON1**, **SYT2**, **THBS3**, **TM2D1**, **TTR**	16	17	Cellular Assembly and Organization, Neurological Disease, Tissue Morphology

* Genes in **BOLD **type are those mapped by the significant genes. ^# ^A score of 3 or greater was considered significant (*p *< 0.001)

**Figure 1:**
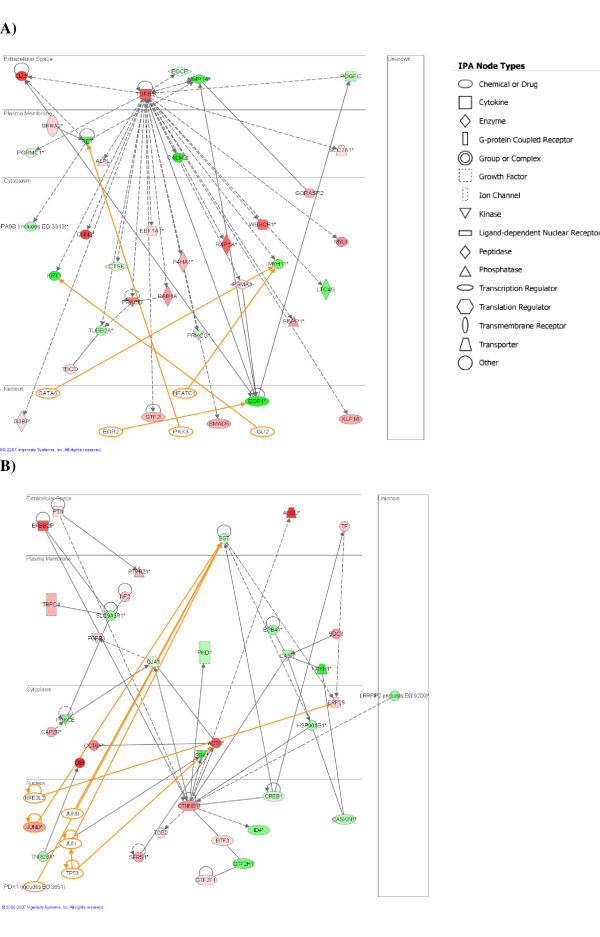
Pathway analysis based on the Ingenuity Pathway Knowledge Base (IPKB). The two highest scoring networks from the PRG list are shown. The shaded notes are genes from among the PRG list; colour shading corresponds to the type of deregulation, red for up-regulated genes, and green for down-regulated. White open nodes are not from among the 349 PRG list genes but are transcription factors that are associated with the regulation of some of these genes identified by IPKB. The shape of the node indicates the major function of the protein. A line denotes binding of the products of the two genes while a line with an arrow denotes 'acts on'. A dotted line denotes an indirect interaction and the orange lines indicate potential interaction with those transcription factors added by IPA that are not on the PRG list.

**Figure 2:**
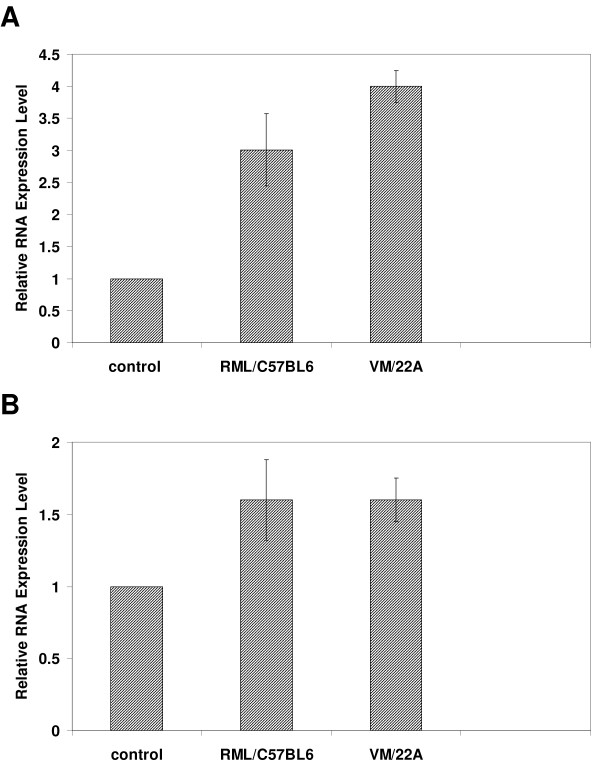
Validation of microarray data by real-time PCR. The levels of expression in two different models of scrapie infected mice (RML/C57BL6 and VM/22A) were measured for two genes (A) TGFB1 and (B) CTNNB1. Expression levels were measured in three individual mice by direct comparison with that from pooled RNA isolated from 3 age-matched, mock-infected, control mice performed in triplicate.

The two highest ranked networks are provided as Figures 1 and 2; each contain 35 PRG's with very strong evidence of connectivity between the genes. The biological functions and/or diseases that were most significant to the genes found in each network were identified. The top ranked network identified by IPA (Figure 1A) is associated with tissue development, specifically the determination of the quantity of cells, particularly phagocytes, and in biological processes controlling cell death and survival. Strikingly, network 1 consists of genes that almost all interact directly with transforming growth factor (TGF)-beta 1. The up-regulation of TGF-beta 1 was confirmed by RT-PCR (Figure 2A). TGF-beta 1 is a multifunctional cytokine that is a potent regulator of injury and inflammatory responses in the central nervous system and has been implicated in cerebral amyloid deposition and pathogenesis of neurodegenerative diseases, including prion diseases [10-13]. TGF-beta 1 expression has been previously reported to increase throughout prion disease concomitant with astrocytes and microglial accumulation [14,15]. Indeed, TGF-beta1 is largely produced in astrocytes and microglia but can be expressed in neurons. Over expression of TGF-beta1 in astrocytes protects adult mice against neurodegeneration during acute, excitotoxic and chronic injury [16], its loss leads to increased neuronal cell death and gliosis [17,18]. Its role during prion induced neurodegeneration is likely to be to suppress inflammation in the brain that could exacerbate the course of the disease.

Network 2 (Figure 1B) contains genes that are primarily involved in nervous system function. The major biological functions of genes in this network are learning and behaviour (SGK, PTN, SDC3, CREB1, EPB41), neuronal migration and outgrowth of neurites (NF2, PRKCE, FGFR3, GJA1, CREB1, PTN, PTPRZ1, SST) and cell cycle progression (SST, CTNNB1, TF, PKD1, TOB2). The genes in this network may play a role in the loss of function and eventual death of neurons that underlies the irreversible pathogenesis of prion induced neurodegenerative diseases.

As in network 1 this network also contains a central gene, CTNNB1 that interacts in some way with a large proportion of the other network genes. The small but consistent up-regulation of CTNNB1 was confirmed by RT-PCR, Figure 2B. This gene is a phosphoprotein involved in Wnt signaling and is an important mediator of cell survival, but has previously not been shown to have a role in prion diseases.

Other up-regulated CTNNB1 interacting genes in this network are SGK, PTN, PTPRZ1 and SDC3. SGK is a serine/threonine kinase, serum and glucocorticoid-regulated kinase 1, which is up-regulated in response to various external stimuli and a number of neurological diseases including. SGK appears to be ubiquitously up-regulated in the brain in response to a strong stress signal and may have a neuroprotective effect like CTNNB1 which is gradually overridden by deleterious mechanisms during the disease process [19]. PTPRZ1 and SDC3 are both receptors expressed on neurons; significantly they share the same ligand, the heparin-binding growth factor PTN, which is also an up-regulated PRG suggesting. Although PTN is known to stimulate neuron and astrocyte differentiation [20,21] the receptors PTPRZ1 and SDC3 are primarily expressed only on neurons. PTN has also been shown to be up-regulated in response to cell stress [22] and to have a neuroprotective role in some instances [23].

### Prediction of cell-specific regulation of PRG's

One of the major pathological features of prion diseases is astrocytosis. As our results were obtained with whole brain tissue it is likely that many deregulated genes were of astrocytic/microglial origins. To give a more complete picture and greater accuracy in the identification of specific pathways involved in prion pathogenesis, we annotated the PRG list based on information on the specific cellular location of the expression of individual genes. To do this we used data from various sources including published microarray studies, PubMed searches and an online *in situ *hybridization database, the Allen Brain Atlas (ABA) [24]. This resulted in the basic annotation of the PRG list in terms of the cell-type; in this case astrocytes/microglia versus neurons. Especially useful was a recent genomic study that identified distinctive transcriptional profiles in astrocytes from in vitro model systems as well as primary astrocytes; these experiments produced a list of 85 astrocyte-specific candidate genes [25]. Comparison of this list with our PRG list revealed that at least one third of these genes were amongst the up-regulated PRG. We used this list, plus further gene expression data from a study by Ponomarev *et al *[26] to compile a list of 61 genes, PRG-A, that we concluded to be exclusively or predominantly expressed in astrocytes or microglia. These genes are provided in Additional File 3. The remainder of the PRG list was named PRG-N and contained some genes known to be expressed in neurons, due to incomplete annotation; however, it is likely that some genes in this list may be expressed in other cell types within the CNS.

**Figure 3:**
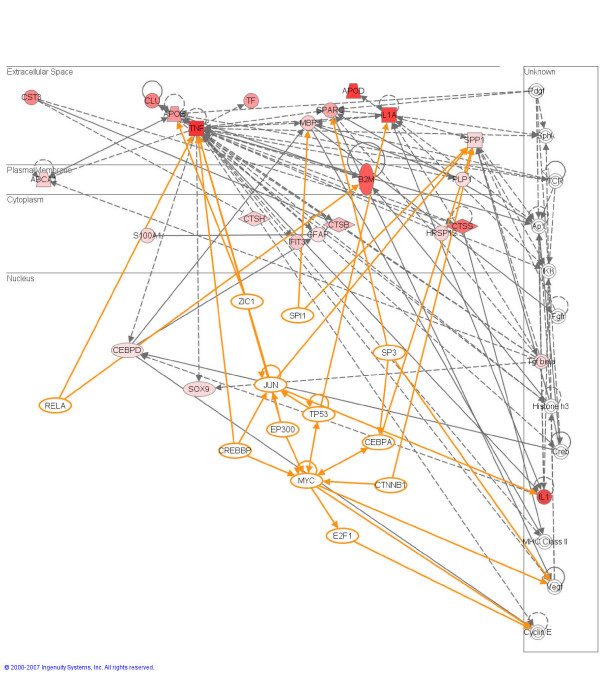
Pathway analysis based on the Ingenuity Pathway Knowledge Base (IPKB). The highest scoring network from the PRG-A list is shown. Annotation is as described for Figure 1.

We analyzed the PRG-A and PRG-N lists in isolation to reveal the most significant biological processes/diseases associated with these sub-groups of genes. Not surprisingly, the PRG-A list contained genes primarily involved in cellular proliferation and growth, cellular migration and inflammatory disease, accurately reflecting the proliferation, activation, and migration of astrocytes and glial cells to damaged areas of the brain. The most significant network is provided in Figure 3, all the PRGs are up-regulated.

Another highly represented group of genes from this list (12/61) have an association with lipid metabolism and transport as annotated in the IPA Knowledge Base database; APOD, APOE, ABCA1, CTSS, TNF, IL1A, MBP, PLP1, PRDX6, CLU, CEBPD, TGFB1. A disruption in lipid metabolism and signaling is one of the early alterations apparent in many neurodegenerative diseases, including prion diseases [27,9]. This group includes some of the most up-regulated PRG genes identified in the study including APOE, APOD and ABCA1 involved in the transport of cholesterol. Neurons require APOE-cholesterol to develop synapses *in vitro*; APOE-cholesterol synthesis occurs mainly in glial cells and is secreted in a process involving ABCA1, a cholesterol efflux pump in the cellular lipid removal pathway. Neurons pick up cholesterol via an LDL receptor. Interestingly, three genes from the PRG-N list were also involved in lipid biosynthesis, specifically cholesterol catabolism, and are down-regulated in our mouse models; DHCR24, HMGCS2 and CYP46A. These genes previously been reported to have an association with Alzheimer's disease [28]. Deregulation of lipid homeostasis, particularly a cholesterol turnover in the CNS, is a feature common to neurodegenerative diseases including prion disease. Indeed cholesterol metabolites are targets of a number of recent studies aimed at the identification of early biomarkers for neurodegenerative diseases [29].

**Figure 4:**
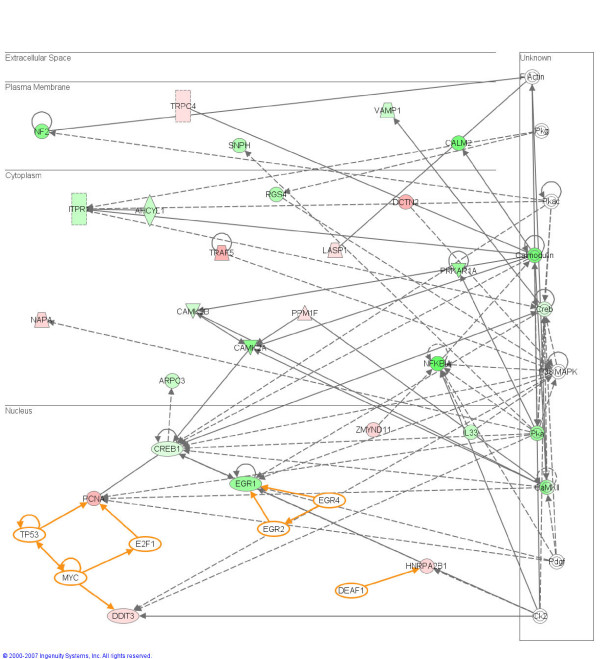
Ingenuity Network analysis showing interactions between PRGs from the PRG-N list, i.e. those genes predominantly expressed non-astrocyte/microglia CNS cells. Annotation is as described for Figure 1.

**Figure 5:**
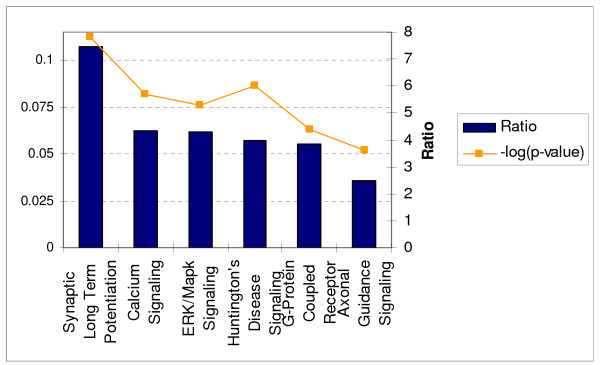
Significant canonical pathways over-represented by PRGs predominantly expressed non-astrocyte/microglia CNS cells as determined using the IPKB functional analysis tool.

A number of mechanisms for neuronal cell death during prion disease have been suggested including the direct toxicity of PrP^Sc ^and mechanisms in which the normal cellular function of PrP^C ^is subverted resulting the triggering of pathways that lead to cell death [30]. In either case it is vital to determine the specific cell death pathways in affected neurons in order to fully understand the disease process and develop drugs that promote neuronal survival. We next used the Ingenuity Pathway Analysis tool to identify biological networks and cellular functions that were most significant to the PRG-N list. The top-ranked pathway is shown in Figure 4 and contains largely down-regulated genes. The most affected biological processes were molecular transport and protein trafficking, regulation of gene expression, cellular movement and cell signalling. Additionally a number of signalling pathways, in particular those involved in synaptic long term potentiation, calcium signalling, and the ERK/MAPK signalling pathway were significantly over represented (p < 0.001) in this group of genes, Figure 5.

All of these pathways have previously been reported to contribute to neuronal death in prion diseases. Abnormalities in synaptic plasticity are believed to be one of the earliest features of prion disease, detectable at about the same time as the deposition of PrP^Sc^, although there are conflicting reports as to the specific electrophysiological changes that take place [31,32]. Significantly, data from many of these electrophysiologic, and Ca^2+ ^measurement studies in neurons converge to suggest that the recruitment of PrP^C ^into prions leads to compromised Ca^2+ ^homeostasis [33-35]. Additionally, these studies, as well as a number of other genomic studies, strongly implicate calcium homeostasis as a biological process that is deregulated in prion diseased brain. We cannot determine from this data whether synapse dysfunction, and alterations in calcium signalling are a cause or effect of neuronal death in prion disease. However, it is known that synaptic dysfunction can render neurons vulnerable to excitotoxicity and apoptosis by a mechanism involving disruption of cellular calcium homeostasis.

**Figure 6:**
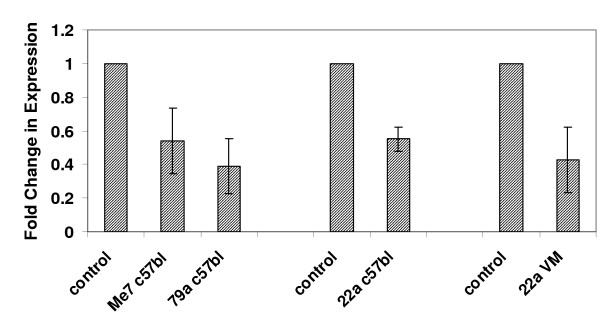
Validation of the level of expression of EGR1 by real-time PCR. The levels of expression in four different models of scrapie infected mice (ME7/C57BL6, 79A/C57BL6, 22A/C57BL6, 22A/VM) were measured. In each case the expression levels were measured in three individual mice by direct comparison with that from pooled RNA isolated from 3 age-matched, mock-infected, control mice performed in triplicate.

In total, 11 genes from the Ingenuity ERK/MAPK signaling pathway were found to be over represented amongst the PRG-N genes; CREB1, H3F3B, PPP2R4, PRKAR1A, PRKCD, PRKCE, PRKCG, RAP1A, TLN1, YWHAB, YWHAG. The ERK/MAPK pathway is stimulated by various growth factors and extracellular stimuli and plays an important role in transducing stress-related signals in eukaryotic cells and promoting differentiation and survival of neuronal cells. A number of previous reports have provided evidence that the deregulation of MAPK pathways play an active role in disease pathogenesis, possibly by upsetting the balance of intracellular signaling in neurons [36,37]. Indeed, major targets of the MAPK pathway are the closely related immediate early genes CREB and EGR1, which are down-regulated PRGs. Both of these genes code for transcriptional regulators known to be phosphorylated during neural activity that leads to long-term synaptic plasticity [38,39] and are thus used as markers of neural activity [40,41]. Previous studies have linked decline in the expression of CREB to a progressive increase of apoptosis [42], and in one study the disruption of CREB1 expression in mouse forebrain, resulted in progressive neurodegeneration in the hippocampus and in the dorsolateral striatum [43]. CREB has also been shown to share a disproportionate number of transcriptional targets with the other down-regulated IEG, EGR1 [44]. EGR1 is one of the most consistently down-regulated PRG genes that we detected in this study; we confirmed its expression in a number of models of mouse scrapie by quantitative RT-PCR at both clinical of infection (Figure 6). There is evidence to suggest that both CREB and EGR are activated by phosphorylation in prion disease [45,46]. This seemingly contrasts with our finding of down-regulation of gene expression; however, sustained activation of regulatory proteins by phosphorylation is often followed by a reduction of gene expression as a mechanism of feedback regulation. Indeed a number of studies have shown that EGR1 is involved in regulation of the MAPK pathway as part of a negative feed back loop; activation of the ERK/MAPK induces active EGR1 which in term transcribes cdk5/p35, that is able to "switch off" the signaling cascade [36,37].

Four of the deregulated genes are activators of the ERK/MAPK signaling pathway; PRKCG, PRKCE, PRKCD and PRKAR1A. All of these genes are down-regulated apart from PRKCD, and three of the four kinases belong to the same family, protein kinase C (PKC). Different PKC isoforms can have opposing roles in particular signaling pathways and can act as sensors mediating either pro- or anti-apoptotic outcomes to different stimuli [47]. PKRCD is a key mediator of apoptosis, and its up-regulation during prion diseases suggests that this PKC isoform may have a role in prion pathogenesis either in gliosis or apoptosis of neurons. Indeed, a number of studies have shown that a damaging insult to brain tissue is able to induce the PRKCD gene in cells such as cortical neurons, astrocytes and in microglia, in which it is not present under physiological conditions [48].

**Figure 7:**
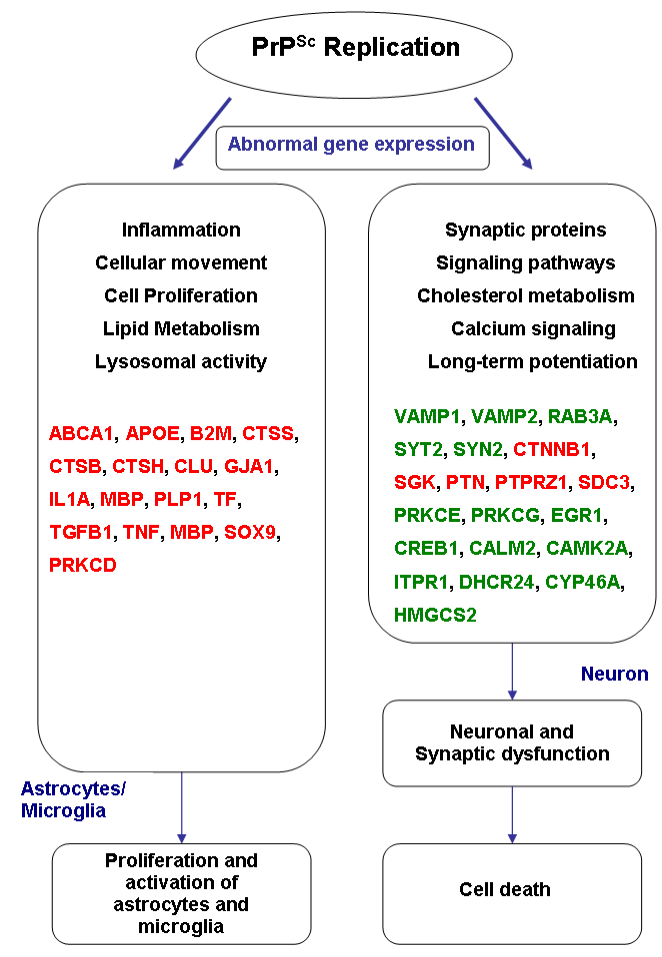
Summary of the genes, pathways and biological processes that characterize the key events in prion pathogenesis. Schematic of the postulated molecular and cellular pathogenic mechanisms triggered during prion-induced neurodegeneration. Genes in red are those significantly up-regulated during disease, while green type indicates down-regulated genes.

A summary of the results from this study showing the genes, pathways and biological processes that characterize the key molecular events in clinically prion infected mouse brain is provided as a schematic in Figure 7.

## Conclusion

Microarray experiments have been performed on tissues affected by many diverse diseases in efforts to document the changes in tissues at the transcriptional level. The majority of such studies generally result in the identification of hundreds of genes that are involved in many different biological processes and pathways. In essence most of these studies provide a very broad overview of transcription in the tissues involved in disease pathogenesis, with little structured information on the specific processes and pathways occurring in individual cells. In this study we used DNA microarray analysis to identify genes that are consistently differentially expressed in multiple scrapie infected mouse models. We went further than our previous study by including data from a number of different models of mouse-adapted scrapie. The large numbers of mice used in the study, and the variety of different mouse models provided greater sensitivity and statistical confidence in selection of differentially expressed genes allowed us to identify a comprehensive list of genes, PRGs, from our BMAP microarrays that are consistently deregulated in these models. We show that many of these PRGs are most likely expressed in proliferating astrocytes and microglia in the brains of prion infected mice.

Although a number of cell types are able to propagate prions it is neuronal cells which sustain the damage which leads to clinical symptoms and death. A recent study by Malluci *et al *showed that the replication of prion in non-neuronal cells is insufficient to cause damage to PrP-null neurons suggesting that deregulated genes expressed in non-neuronal cells are secondary to those involved in neuronal damage [49]. However, a complimentary study by Jeffrey *et al *in which transgenic mice expressing PrP^C ^only in astrocytes did succumb to disease, albeit following an extended incubation period, suggests that the mechanisms of cell death are complicated, and not restricted to a neuronal response [50]. It is possible that different pathways leading to cell death are triggered either by the direct effect of a build up of PrP^Sc ^or by an indirect effect triggered by the intra-neuronal replication of PrP^Sc^. The implementation of gene expression studies in isolated cell populations to identify pathways triggered in these different models will be instrumental in determining the molecular events that define prion pathogenesis *in vivo*.

In this report we show a bioinformatic strategy to separate non-neuronal and neuronal gene expression. Although many of this core group of genes has been identified in previous genomic studies of neurodegeneration in mouse-adapted scrapie, the use of these new bioinformatics tools allows us to gain further insights into the prion pathobiology. Further work using microdissection strategies and single cell analysis is essential to identify those genes most important to pathogenesis.

## Methods

### Biological material

C57BL/6 or VM mice were inoculated intracerebrally with 20 μl of 1% brain homogenate from mice infected with scrapie strains 22A, ME7, or 79a. At the onset of clinical symptoms 8 to 10 mice per sample group were sacrificed and the whole brains used for RNA extraction. An equal number of age-matched, mock-infected mice (inoculated with a 1% brain homogenate of either C57BL/6 or VM mice) were used as controls for each group. Due to the long time course of infection in mice we stored mouse brain tissue at -80°C in RNAlater solution (Ambion) until all experimental time points were completed. All procedures involving live animals were approved by the Canadian Science Centre for Human and Animal Health Animal Care Committee.

### Microarray analysis

RNA was isolated from brain tissue by disruption in Trizol reagent (Invitrogen) and was further purified using RNeasy columns (Qiagen) according to the manufacturer's instructions. A quantity of 10 μg of total RNA was used as a template for oligo dT primed reverse transcription which incorporated amino allyl-dUTP (Sigma) into the cDNA. The remaining RNA template was hydrolyzed and the cDNA product purified using the QIAquick PCR purification system (Qiagen). PCR products were dried down, then resuspended in 0.1 M Na_2_CO_3 _buffer, and incubated for 1 hour in either an Alexa Fluor^555 ^(AF555) or Alexa Fluor^647 ^(AF647) mono-functional reactive dye (Molecular Probes) made up in DMSO. Labelled cDNA was also purified using QIAquick PCR purification columns.

### Array hybridization and scanning

Labelled cDNA was dried down and resuspended in 60 μl DIG Easy Hyb hybridization buffer (Roche) containing calf thymus DNA (Sigma) and yeast tRNA (Life Technologies). The labelled cDNAs were applied to arrays that contained 11,136 cDNAs from the Brain Molecular Anatomy Project (BMAP). Arrays were constructed in-house as previously described [2]. Hybridization was performed under M Series Lifter Slips (Erie Scientific Company) and the slides incubated in a hybridization chamber at 42°C for 16 hours. Following hybridization lifter slips were removed and the arrays washed a total of 3 times for 5 minutes each in 1 × SSC, 0.2% SDS then 0.1 × SSC, 0.2% SDS pre-warmed to 42°C. A final wash in 0.1 × SSC at room temperature was performed and slides spun to dry. Spots were quantitated and background signals removed using Array-Pro^® ^Analyser software (Media Cybernetics, Inc).

Data were stored and normalized in the GeneTraffic Microarray Database and Analysis System (Iobion Informatics, La Jolla, CA). The raw data was filtered so that individual spots had to pass a number of quality criteria including, minimum intensity levels, minimum signal to background ratios, and no greater than 10% missing values for each gene. Genes who passed these criteria were used for further data analysis. Each slide was then normalized using a linear regression, smoothing algorithm (Loess best-fit) over individual array sub grids. Log^2 ^ratios were used for statistical analysis using EDGE (Extraction of Differential Gene Expression), an open source software program for the significance analysis of DNA microarray experiments [51]. Significance calculations are based on the Optimal Discovery Procedure developed by Storey et al [51], which estimates the optimal rule for identifying differentially expressed genes [52,53]. A significance measure is assigned to each gene *P*-value cut-off of 0.05 was selected as the significance threshold by which significantly differentially expressed genes were identified.

### Real time PCR

To remove any contaminating genomic DNA from the RNA template approximately 2 μg of total RNA was DNase I treated using the DNA-free system (Ambion). TaqMan^® ^quantitative real-time PCR was performed on cDNA reverse transcribed from total RNA collected from brain tissue samples with several TaqMan^® ^Gene Expression Assays, and all protocols and analysis were followed as per the manufacturer (Applied Biosystems). Reverse transcription of 100 ng of total RNA was carried out using the High Capacity cDNA Reverse Transcription kit. Subsequently, 1 μl from each reverse transcription reaction was assayed in a 20 μl singleplex reaction for real-time quantitation with the PCR 7500 Fast System (Applied Biosystems). Each sample was run in biological triplicate, of which 3 technical replicates were performed. GAPDH was used as the endogenous control, and gene expression of target genes for infected samples were quantitatively measured relative to those of RNA from mock-infected, age matched control mice. Relative quantification values were determined using the 2^-ΔΔct ^method, and expressed as fold-change in the infected mice versus mock-infected controls.

### Functional analysis of the data set

Annotation of expression data was performed with reference to a number of sources which include; NIH DAVID the Gene Ontology Consortium, Kyoto Encyclopedia of Genes and Genomes (KEGG) pathway [54-56]. The Ingenuity Pathway Analysis functional analysis tool was also used to identify the biological functions and/or diseases that were most significant to the data set. Genes from the dataset were associated with biological functions and/or diseases. Fischer's exact test was used to calculate a p-value determining the probability that each biological function and/or disease assigned to that data set is due to chance alone. Canonical pathways were also identified from the Ingenuity Pathways Analysis library; canonical pathways that were most significant to the data set were selected. The significance of the association between the data set and the canonical pathway was measured in 2 ways: 1) a ratio of the number of genes from the data set that map to the pathway divided by the total number of genes that map to the canonical pathway is displayed. 2) Fischer's exact test was used to calculate a p-value determining the probability that the association between the genes in the dataset and the canonical pathway is explained by chance alone.

### Network generation

Data containing gene identifiers for the PRG genes and corresponding expression values were uploaded into in the application. Each gene identifier was mapped to its corresponding gene object in the Ingenuity Pathways Knowledge Base. Genes present in this database, called Focus Genes, were overlaid onto a global molecular network developed from information contained in the Ingenuity Pathways Knowledge Base. Networks of these Focus Genes were then algorithmically generated based on their connectivity. Functional analysis was performed on these networks to identify the biological functions and/or diseases that were most significant to the genes in the network. Fischer's exact test was used to calculate a p-value determining the probability that each biological function and/or disease assigned to that network is due to chance alone.

Genes or gene products are represented as nodes, and the biological relationship between two nodes is represented as an edge (line). All edges are supported by at least 1 reference from the literature, from a textbook, or from canonical information stored in the Ingenuity Pathways Knowledge Base. Human, mouse, and rat orthologs of a gene are stored as separate objects in the Ingenuity Pathways Knowledge Base, but are represented as a single node in the network. The intensity of the node color indicates the degree of up- (red) or down- (green) regulation. Nodes are displayed using various shapes that represent the functional class of the gene product. Edges are displayed with various labels that describe the nature of the relationship between the nodes (e.g., P for phosphorylation, T for transcription).

### Accession numbers

The BMAP and Agilent microarray related data were submitted to Gene Expression Omnibus (GEO) under accession number: [GSE10310]

## Abbreviations

Transmissible spongiform encephalopathy (TSE), prion-related gene (PRG), disease-specific protease-resistant prion protein (PrPSc)

## Authors' contributions

GS contributed to data analysis and BMAP cDNA microarray preparation, as well as performing the BMAP microarrays, and developed and maintained the data resource. SM performed all the qRT-PCR procedures and the Agilent microarrays. DP contributed to cDNA microarray preparation and the gene expression analysis. CP and CR contributed to material preparation. SB conceived, led and coordinated the project, performed the data analysis and drafted the manuscript. All authors read and approved the final manuscript.

## Supplementary Material

Additional file 1 Hierarchical clustering plot of log^2 ^ratios for all BMAP genes for four models of mouse adapted scrapie. Although there are some strain specific differences the pattern of gene expression is for the most part similar between the different models. Click here for file

Additional file 2 PRG list with 78 genes implicated in neurological disease marked. Click here for file

Additional file 3 PRG-A list. Candidate PRG genes predominantly expressed in astrocytes or microglia based on profiling experiments performed by Bachoo *et al *and Ponomarev *et al *[25,26] Click here for file
