# Supplementary figures and images for: Comprehensive transcriptional profiling of prion infection in mouse models reveals networks of responsive genes
Source: BMC Genomics. 2008 Mar 3;9:114. doi: 10.1186/1471-2164-9-114 (PMC2294129; doi:10.1186/1471-2164-9-114)

**ME7/C57BL6 79A/ C57BL6 22A/C57BL6 22A/VM**


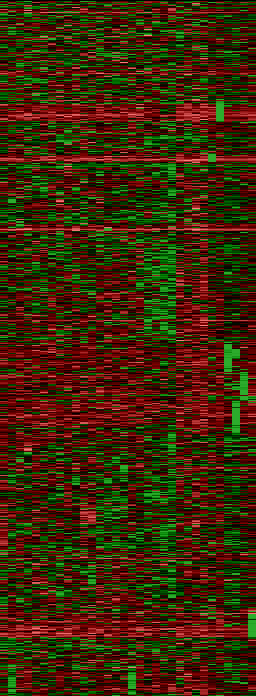

Supplement: Additional file 1 — Hierarchical clustering plot of log2 ratios for all BMAP genes for four models of mouse adapted scrapie. Although there are some strain specific differences the pattern of gene expression is for the most part similar between the different models. [file 1471-2164-9-114-S1.doc]
