# Supplementary material for: Comprehensive transcriptional profiling of prion infection in mouse models reveals networks of responsive genes
Source: BMC Genomics. 2008 Mar 3;9:114. doi: 10.1186/1471-2164-9-114 (PMC2294129; doi:10.1186/1471-2164-9-114)
Supplement: Additional file 2 — PRG list with 78 genes implicated in neurological disease marked. [file 1471-2164-9-114-S2.doc]

**PRG list with 78 genes implicated in neurological disease marked.**

| **Up-regulated genes** | | | **P-Value** | **Average Fold Change** | **Implicated in**  **neurological disease** |
| --- | --- | --- | --- | --- | --- |
| A2BP1* | ataxin 2-binding protein 1 | AI852887 | 0 | 1.21 |  |
| **ABCA1*** | ATP-binding cassette, sub-family A (ABC1), member 1 | AI845514 | 0 | 1.47 | * |
| **ADAM8**  **ANXA3*** | ADAM metallopeptidase domain 8  annexin A3 | AI838487 | 0.006 | 1.35 |  |
| **APLP1*** | amyloid beta (A4) precursor-like protein 1 | AI838490 | 0 | 1.25 | * |
| **APOD*** | apolipoprotein D | AI840024 | 0 | 4.49 | * |
| **APOE*** | apolipoprotein E | AI848248 | 0 | 2.21 | * |
| **ARF4** | ADP-ribosylation factor 2 | AI839821 | 0.065 | 1.31 |  |
| ASRGL1 | asparaginase like 1 | AI847958 | 0.001 | 1.33 |  |
| ATP1B2 | ATPase, Na+/K+ transporting, beta 2 polypeptide | AI845260 | 0 | 1.50 |  |
| ATXN2 | ataxin 2 | AI851011 | 0.081 | 1.17 | * |
| **B2M*** | beta-2-microglobulin | AI848245 | 0 | 3.56 | * |
| **BMPR1B** | bone morphogenetic protein receptor, type IB | AI854816 | 0.009 | 1.35 | * |
| BTF3 | basic transcription factor 3 | AI849189 | 0.005 | 1.19 |  |
| C14ORF11^ | chromosome 14 open reading frame 11 | AI843931 | 0.034 | 1.22 |  |
| **CAPZB*** | capping protein (actin filament) muscle Z-line, beta | AI842317 | 0.074 | 1.20 |  |
| CBLB | Cas-Br-M (murine) ecotropic retroviral transforming sequence b | AI851073 | 0.005 | 1.15 | * |
| **CCT6A** | chaperonin containing TCP1, subunit 6A (zeta 1) | AI838255 | 0 | 1.27 |  |
| CD47 | CD47 molecule | AI853695 | 0 | 1.30 | * |
| **CD9*** | CD9 molecule | AI854515 | 0 | 2.09 | * |
| **CEBPD*** | CCAAT/enhancer binding protein (C/EBP), delta | AI843497 | 0 | 1.23 | * |
| CEP350 | centrosomal protein 350kDa | AI851218 | 0 | 1.53 |  |
| CLEC11A | C-type lectin domain family 11, member A | AI841926 | 0.007 | 1.21 | * |
| **CLU*** | clusterin | AI836624 | 0 | 2.68 | * |
| **CNKSR2** | connector enhancer of kinase suppressor of Ras 2 | AI854548 | 0.022 | 1.26 |  |
| **CNN3** | calponin 3, acidic | AI843550 | 0 | 2.07 |  |
| CRSP2^ | cofactor required for Sp1 transcriptional activation, subunit 2, 150kDa | AI839384 | 0 | 2.38 |  |
| **CRYAB** | crystallin, alpha B | AI848067 | 0 | 1.39 |  |
| **CST3*** | cystatin C (amyloid angiopathy and cerebral hemorrhage) | AI836882 | 0 | 2.55 | * |
| **CTNNB1** | catenin (cadherin-associated protein), beta 1, 88kDa | AI849257 | 0.061 | 1.35 | * |
| **CTSB*** | cathepsin B | AI838658 | 0 | 1.67 | * |
| CTSH* | cathepsin H | AI840178 | 0 | 1.45 |  |
| CTSK | cathepsin K (pycnodysostosis) | AI838980 | 0.015 | 1.27 |  |
| **CTSS*** | cathepsin S | AI845967 | 0 | 3.05 | * |
| CYB5A | cytochrome b5 type A (microsomal) | AI854779 | 0.005 | 1.27 |  |
| **DBI*** | diazepam binding inhibitor (GABA receptor modulator, acyl-Coenzyme A binding protein) | AI841459 | 0 | 1.70 | * |
| **DCTN2** | dynactin 2 (p50) | AI838204 | 0 | 1.61 | * |
| **DDIT3** | DNA-damage-inducible transcript 3 | AI840919 | 0.0273 | 1.24 |  |
| **DHRS1*** | dehydrogenase/reductase (SDR family) member 1 | AI845580 | 0 | 1.95 |  |
| **EEF1A1*** | eukaryotic translation elongation factor 1 alpha 1 | AI835939 | 0.001 | 1.59 |  |
| EIF2AK1 | eukaryotic translation initiation factor 2-alpha kinase 1 | AI842443 | 0.003 | 1.27 |  |
| EPB49* | erythrocyte membrane protein band 4.9 (dematin) | AI835123 | 0.001 | 1.50 |  |
| **ERBB2IP** | erbb2 interacting protein | AI843677 | 0 | 1.60 |  |
| ERP29 | endoplasmic reticulum protein 29 | AI851833 | 0.077 | 1.10 |  |
| EXOSC9 | exosome component 9 | AI851373 | 0.008 | 1.23 |  |
| **FAIM2** | Fas apoptotic inhibitory molecule 2 | AI847680 | 0.001 | 1.15 |  |
| FAU | Finkel-Biskis-Reilly murine sarcoma virus (FBR-MuSV) ubiquitously expressed (fox derived); ribosomal protein S30 | AI847325 | 0.006 | 1.27 |  |
| FTH1* | ferritin, heavy polypeptide 1 | AI835905 | 0 | 1.53 |  |
| **FXYD*1** | FXYD domain containing ion transport regulator 1 (phospholemman) | AI853335 | 0 | 2.22 |  |
| **GART** | phosphoribosylglycinamide formyltransferase, phosphoribosylglycinamide synthetase, phosphoribosylaminoimidazole synthetase | AI852195 | 0.093 | 1.23 |  |
| **GFAP*** | glial fibrillary acidic protein | AI836096 | 0.0403 | 1.21 | * |
| **GFRA2** | GDNF family receptor alpha 2 | AI843391 | 0.03 | 1.24 | * |
| **GJA1** | gap junction protein, alpha 1, 43kDa (connexin 43) | AI852313 | 0 | 1.36 | * |
| GLUD1 | glutamate dehydrogenase 1 | AI852301 | 0.004 | 1.34 |  |
| **GNB4** | guanine nucleotide binding protein (G protein), beta polypeptide 4 | AI853415 | 0 | 3.38 |  |
| **GNG5** | guanine nucleotide binding protein (G protein), gamma 5 | AI843937 | 0 | 1.45 |  |
| **GORASP2** | golgi reassembly stacking protein 2, 55kDa | AI835585 | 0 | 1.37 |  |
| **GPT2** | glutamic pyruvate transaminase (alanine aminotransferase) 2 | AI843941 | 0.001 | 1.23 |  |
| **GRN*** | granulin | AI846605 | 0 | 1.38 | * |
| **GSTA3** | glutathione S-transferase A3 | AI843721 | 0.002 | 1.28 |  |
| GTF2I | general transcription factor II, i | AI844540 | 0.001 | 1.41 |  |
| HDAC11 | histone deacetylase 11 | AI835406 | 0 | 1.73 |  |
| **HEXB*** | hexosaminidase B (beta polypeptide) | AI854206 | 0 | 2.90 | * |
| HIPK2 | homeodomain interacting protein kinase 2 | AI846656 | 0.004 | 1.22 | * |
| HNRPA2B1 | heterogeneous nuclear ribonucleoprotein A2/B1 | AI844131 | 0.001 | 1.28 |  |
| **HRSP12*** | heat-responsive protein 12 | AI850467 | 0 | 1.41 | * |
| HSPA9B | heat shock 70kDa protein 9B (mortalin-2) | AI849368 | 0 | 1.28 |  |
| **IFIT3** | interferon-induced protein with tetratricopeptide repeats 3 | AI854552 | 0 | 1.53 |  |
| **IFITM3** | interferon induced transmembrane protein 3 (1-8U) | AI846398 | 0.007 | 1.34 |  |
| **IGBP1** | immunoglobulin (CD79A) binding protein 1 | AI853091 | 0.014 | 1.35 |  |
| **IGF2** | insulin-like growth factor 2 (somatomedin A) | AI841331 | 0 | 1.17 | * |
| **IL1A*** | Interleukin 1A |  |  |  | (TaqMan) |
| **ITM2C** | integral membrane protein 2C | AI839869 | 0 | 1.30 |  |
| **KCTD1** | potassium channel tetramerisation domain containing 1 | AI838815 | 0.097 | 1.18 |  |
| **KLF16** | Kruppel-like factor 16 | AI843742 | 0.001 | 1.28 |  |
| **LAMP2*** | lysosomal-associated membrane protein 2 | AI850263 | 0 | 1.35 |  |
| **LAPTM4A** | lysosomal-associated protein transmembrane 4 alpha | AI846864 | 0.002 | 1.34 |  |
| **LAPTM5*** | lysosomal associated multispanning membrane protein 5 | AI854633 | 0.008 | 1.67 | * |
| LASP1 | LIM and SH3 protein 1 | AI843826 | 0.004 | 1.00 |  |
| **LMNA** | lamin A/C | AI845319 | 0 | 1.43 |  |
| LR8^ | LR8 protein | AI851466 | 0 | 1.60 |  |
| **LY6C***  **LY86** | lymphocyte antigen 6 complex, locus C  lymphocyte antigen 86 | AI844280  AI843659 | 0  0 | 1.60  2.16 | * |
| **MAP1LC3B** | microtubule-associated protein 1 light chain 3 beta | AI841311 | 0.003 | 1.17 |  |
| **MAT2A** | methionine adenosyltransferase II, alpha | AI850268 | 0.001 | 1.53 |  |
| MBP | myelin basic protein | AI839613 | 0.002 | 1.48 | * |
| **MCOLN1** | mucolipin 1 | AI841374 | 0.001 | 1.68 |  |
| MLYCD | malonyl-CoA decarboxylase | AI834893 | 0 | 1.36 | * |
| MYL6 | myosin, light polypeptide 6, alkali, smooth muscle and non-muscle | AI835907 | 0.008 | 1.23 |  |
| NACA | nascent-polypeptide-associated complex alpha polypeptide | AI836995 | 0.01 | 1.26 |  |
| **NAPA** | N-ethylmaleimide-sensitive factor attachment protein, alpha | AI849260 | 0.006 | 1.29 |  |
| NDUFA2 | NADH dehydrogenase (ubiquinone) 1 alpha subcomplex, 2, 8kDa | AI851603 | 0.008 | 1.31 |  |
| NSBP1 | nucleosomal binding protein 1 | AI840555 | 0.006 | 1.22 |  |
| OSBPL1A | oxysterol binding protein-like 1A | AI840647 | 0.014 | 1.31 |  |
| PCNA | proliferating cell nuclear antigen | AI849847 | 0 | 1.55 |  |
| **PGCP** | plasma glutamate carboxypeptidase | AI852019 | 0.007 | 1.24 |  |
| **PGRMC1** | progesterone receptor membrane component 1 | AI843194 | 0.016 | 1.43 |  |
| **PIGS** | phosphatidylinositol glycan anchor biosynthesis, class S | AI848040 | 0 | 1.60 |  |
| PLP1 | proteolipid protein 1 (Pelizaeus-Merzbacher disease, spastic paraplegia 2, uncomplicated) | AI839745 | 0.029 | 1.31 | * |
| **PMP22** | peripheral myelin protein 22 | AI851107 | 0.012 | 1.36 | * |
| POLR2F | polymerase (RNA) II (DNA directed) polypeptide F | AI835726 | 0.003 | 1.31 |  |
| POLR2G* | polymerase (RNA) II (DNA directed) polypeptide G | AI845081 | 0 | 1.34 |  |
| POMP | proteasome maturation protein | AI850108 | 0.002 | 1.26 |  |
| PPM1F | protein phosphatase 1F (PP2C domain containing) | AI842786 | 0 | 1.17 |  |
| **PPT1*** | palmitoyl-protein thioesterase 1 (ceroid-lipofuscinosis, neuronal 1, infantile) | AI850126 | 0 | 1.28 |  |
| PRKCD* | protein kinase C, delta | AI843656 | 0.003 | 1.29 | * |
| PSMA3 | proteasome (prosome, macropain) subunit, alpha type, 3 | AI843948 | 0.001 | 1.25 |  |
| PTN | pleiotrophin (heparin binding growth factor 8, neurite growth-promoting factor 1) | AI846004 | 0.001 | 1.32 | * |
| PTPMT1 | protein tyrosine phosphatase, mitochondrial 1 | AI841892 | 0.005 | 1.50 |  |
| PTPN5 | protein tyrosine phosphatase, non-receptor type 5 (striatum-enriched) | AI836512 | 0.004 | 1.20 |  |
| **PTPRZ1** | protein tyrosine phosphatase | AI849177 | 0.001 | 1.39 | * |
| **RAB11B** | RAB11B, member RAS oncogene family | AI843646 | 0.001 | 1.21 |  |
| **RAB1A** | RAB1A, member RAS oncogene family | AI850349 | 0 | 1.63 |  |
| **RAB31** | RAB31, member RAS oncogene family | AI850865 | 0 | 1.34 |  |
| RAD17 | RAD17 homolog (S. pombe) | AI846300 | 0.001 | 1.46 |  |
| RAP1A | RAP1A, member of RAS oncogene family | AI835434 | 0 | 1.52 |  |
| **RAP1B** | RAP1B, member of RAS oncogene family | AI835947 | 0.042 | 1.18 |  |
| RASA2 | RAS p21 protein activator 2 | AI844397 | 0 | 1.32 |  |
| RHOF | ras homolog gene family, member F (in filopodia) | AI845056 | 0 | 3.60 |  |
| RPL10A* | ribosomal protein L10a | AI849117 | 0 | 1.44 |  |
| **RPL11** | ribosomal protein L11 | AI850501 | 0.001 | 1.26 |  |
| RPL23 | ribosomal protein L23 | AI851588 | 0.036 | 1.37 |  |
| RPL24 | ribosomal protein L24 | AI839914 | 0.002 | 1.48 |  |
| RPL26 | ribosomal protein L26 | AI834863 | 0.09 | 1.37 |  |
| RPL37A | ribosomal protein L37a | AI837822 | 0.004 | 1.27 |  |
| RPL4 | ribosomal protein L4 | AI849118 | 0 | 1.28 |  |
| RPL9* | ribosomal protein L9 | AI848697 | 0.001 | 1.26 |  |
| RPS27 | ribosomal protein S27 (metallopanstimulin 1) | AI854238 | 0 | 1.41 |  |
| RPS3 | ribosomal protein S3 | AI844945 | 0.001 | 1.30 |  |
| **RPS9** | ribosomal protein S9 | AI838295 | 0.003 | 1.61 |  |
| **RTN4*** | reticulon 4 | AI842469 | 0 | 1.20 | * |
| **RTN4R*** | reticulon 4 receptor | AI844168 | 0 | 1.22 | * |
| **S100A1*** | S100 calcium binding protein A1 | AI841575 | 0.095 | 1.37 |  |
| **SAP30** | Sin3A-associated protein, 30kDa | AI849299 | 0 | 1.59 |  |
| SCXA^ | scleraxis homolog A (mouse) | AI845863 | 0 | 1.34 |  |
| **SDC3*** | syndecan 3 (N-syndecan) | AI854015 | 0.005 | 1.15 |  |
| **SECISBP2** | SECIS binding protein 2 | AI850227 | 0.068 | 1.33 |  |
| **SEL1L** | sel-1 suppressor of lin-12-like (C. elegans) | AI849408 | 0.02 | 1.46 |  |
| SET | SET translocation (myeloid leukemia-associated) | AI844542 | 0.005 | 1.27 |  |
| **SEZ6L2** | seizure related 6 homolog (mouse)-like 2 | AI835913 | 0 | 1.33 |  |
| SFRS1 | splicing factor, arginine/serine-rich 1 (splicing factor 2, alternate splicing factor) | AI842817 | 0.005 | 1.17 |  |
| SGCB | sarcoglycan, beta (43kDa dystrophin-associated glycoprotein) | AI844132 | 0.039 | 1.30 |  |
| **SGK** | serum/glucocorticoid regulated kinase | AI849519 | 0.022 | 1.55 |  |
| SGPP1 | sphingosine-1-phosphate phosphatase 1 | AI847595 | 0 | 1.39 |  |
| SHFM1 | split hand/foot malformation (ectrodactyly) type 1 | AI849481 | 0.03 | 1.26 |  |
| SIN3B | SIN3 homolog B, transcription regulator (yeast) | AI834948 | 0.003 | 1.04 |  |
| SLC37A4 | solute carrier family 37 (glycerol-6-phosphate transporter), member 4 | AI845186 | 0.008 | 1.29 |  |
| SNX17 (includes EG:9784) | sorting nexin 17 | AI836136 | 0.023 | 1.44 |  |
| **SNX5** | sorting nexin 5 | AI840437 | 0.093 | 1.36 |  |
| **SOX9*** | SRY (sex determining region Y)-box 9 (campomelic dysplasia, autosomal sex-reversal) | AI852411 | 0.002 | 1.35 | * |
| **SPARC*** | secreted protein, acidic, cysteine-rich (osteonectin) | AI845741 | 0 | 1.96 | * |
| SPG20 | spastic paraplegia 20, spartin (Troyer syndrome) | AI840044 | 0 | 1.61 |  |
| **SPP1*** | secreted phosphoprotein 1 (osteopontin, bone sialoprotein I, early T-lymphocyte activation 1) | AI847805 | 0.001 | 1.37 | * |
| SRP9 | signal recognition particle 9kDa | AI851872 | 0.094 | 1.34 |  |
| **TF*** | transferrin | AI841326 | 0.085 | 2.17 | * |
| **TGFB1** | transforming growth factor, beta 1 (Camurati-Engelmann disease) | AI838343 | 0.002 | 1.35 | * |
| TIMM50 | translocase of inner mitochondrial membrane 50 homolog (S. cerevisiae) | AI838447 | 0.075 | 1.25 |  |
| TM2D1 | TM2 domain containing 1 | AI854293 | 0 | 1.54 |  |
| TM7SF3 | transmembrane 7 superfamily member 3 | AI845077 | 0 | 1.77 |  |
| **TMSB4X** | thymosin, beta 4, X-linked | AI835403 | 0.029 | 1.42 |  |
| TNF | Tumor necrosis factor |  |  |  | (TaqMan) |
| TRAF5 | TNF receptor-associated factor 5 | AI851866 | 0 | 1.59 |  |
| TRPC4 | transient receptor potential cation channel, subfamily C, member 4 | AI836242 | 0.001 | 1.15 |  |
| **TTR*** | transthyretin (prealbumin, amyloidosis type I) | AI848407 | 0 | 1.36 | * |
| TXNDC9 | thioredoxin domain containing 9 | AI850320 | 0 | 1.32 |  |
| UCHL5 | ubiquitin carboxyl-terminal hydrolase L5 | AI838853 | 0.084 | 1.46 |  |
| UGP2 | UDP-glucose pyrophosphorylase 2 | AI851321 | 0.003 | 1.40 |  |
| WBSCR1^ | Williams-Beuren syndrome chromosome region 1 | AI848265 | 0 | 1.45 |  |
| **ZMYND11** | zinc finger, MYND domain containing 11 | AI849660 | 0 | 1.30 |  |
| ZMYND19 | zinc finger, MYND-type containing 19 | AI846307 | 0.001 | 1.30 |  |

| **Down-regulated genes** | | | **P-Value** | **Average Fold Change** | **Implicated in neurological disease** |
| --- | --- | --- | --- | --- | --- |
| ADRA1D | adrenergic, alpha-1D-, receptor | AI839485 | 0.003 | -1.20 | * |
| AES | amino-terminal enhancer of split | AI836421 | 0.015 | -1.33 |  |
| AHCYL1* | S-adenosylhomocysteine hydrolase-like 1 | AI853700 | 0.007 | -1.23 |  |
| ALS2CR2 | amyotrophic lateral sclerosis 2 (juvenile) chromosome region, candidate 2 | AI853959 | 0.036 | -1.31 |  |
| **ARPC3*** | actin related protein 2/3 complex, subunit 3, 21kDa | AI851740 | 0.068 | -1.22 |  |
| ATP6V1A | ATPase, H+ transporting, lysosomal 70kDa, V1 subunit A | AI846190 | 0.001 | -1.22 |  |
| BCAT1 | branched chain aminotransferase 1, cytosolic | AI842819 | 0 | -1.29 |  |
| **BSG** | basigin (Ok blood group) | AI842086 | 0 | -1.24 | * |
| BTBD14B | BTB (POZ) domain containing 14B | AI851205 | 0.093 | -2.31 |  |
| C9ORF26^ | chromosome 9 open reading frame 26 (NF-HEV) | AI854495 | 0.018 | -1.23 |  |
| CA8 | carbonic anhydrase VIII | AI838156 | 0 | -1.27 |  |
| CALM2* | calmodulin 2 (phosphorylase kinase, delta) | AI843756 | 0.002 | -1.65 |  |
| CALM3* | calmodulin 3 (phosphorylase kinase, delta) | AI837642 | 0 | -1.36 |  |
| CAMK2A* | calcium/calmodulin-dependent protein kinase (CaM kinase) II alpha | AI843440 | 0.002 | -1.53 | * |
| **CAMK2B*** | calcium/calmodulin-dependent protein kinase (CaM kinase) II beta | AI849170 | 0 | -1.19 |  |
| **CANT1** | calcium activated nucleotidase 1 | AI840155 | 0 | -1.29 |  |
| CART^ | cocaine- and amphetamine-regulated transcript | AI854310 | 0.001 | -1.48 |  |
| **CASK** | calcium/calmodulin-dependent serine protein kinase (MAGUK family) | AI842158 | 0.002 | -1.13 |  |
| **CASKIN1** | CASK interacting protein 1 | AI845923 | 0 | -1.25 |  |
| CBLN1 | cerebellin 1 precursor | AI839783 | 0.011 | -1.16 |  |
| CCNC | cyclin C | AI854634 | 0.005 | -1.20 |  |
| CHRNB4 | cholinergic receptor, nicotinic, beta 4 | AI845506 | 0.006 | -1.15 | * |
| **CREB1** | cAMP responsive element binding protein 1 | AI837343 | 0.003 | -1.12 | * |
| CYP46A1 | Cytochrome P450, family 46, subfamily A, polypeptide 1 | AI838822 | 0.003 | -1.27 |  |
| **DHCR24** | 24-dehydrocholesterol reductase | AI840156 | 0.033 | -1.25 |  |
| DNAJA1* | DnaJ (Hsp40) homolog, subfamily A, member 1 | AI853868 | 0.02 | -1.20 |  |
| DNER | delta-notch-like EGF repeat-containing transmembrane | AI839243 | 0 | -1.41 |  |
| **DOCK3** | dedicator of cytokinesis 3 | AI840717 | 0.023 | -1.23 |  |
| DPYSL2 | dihydropyrimidinase-like 2 | AI845631 | 0.035 | -1.24 |  |
| **EDA** | ectodysplasin A | AI846651 | 0.014 | -1.16 |  |
| EGR1* | early growth response 1 | AI849001 | 0.097 | -1.45 | * |
| ELAVL2 | ELAV (embryonic lethal, abnormal vision, Drosophila)-like 2 (Hu antigen B) | AI837658 | 0.094 | -1.38 |  |
| FAIM | Fas apoptotic inhibitory molecule | AI843459 | 0.028 | -1.33 |  |
| FALZ^ | fetal Alzheimer antigen | AI848250 | 0.008 | -1.19 |  |
| GPR6 | G protein-coupled receptor 6 | AI852874 | 0.003 | -1.29 |  |
| HIP2 | huntingtin interacting protein 2 | AI848375 | 0.001 | -1.40 |  |
| **HMGCS2*** | 3-hydroxy-3-methylglutaryl-Coenzyme A synthase 2 (mitochondrial) | AI834962 | 0.076 | -1.31 |  |
| **HS3ST2** | heparan sulfate (glucosamine) 3-O-sulfotransferase 2 | AI844694 | 0.001 | -1.29 |  |
| **HSP90AB1*** | heat shock protein 90kDa alpha (cytosolic), class B member 1 | AI848744 | 0.003 | -1.19 |  |
| **HSPB3** | heat shock 27kDa protein 3 | AI844863 | 0.091 | -1.16 |  |
| ID4* | inhibitor of DNA binding 4, dominant negative helix-loop-helix protein | AI843328 | 0.089 | -1.16 |  |
| **ITPR1*** | inositol 1,4,5-triphosphate receptor, type 1 | AI848073 | 0.011 | -1.23 |  |
| **KRT1** | keratin 1 (epidermolytic hyperkeratosis) | AI840489 | 0.003 | -1.41 |  |
| **LATS2** | LATS, large tumor suppressor, homolog 2 (Drosophila) | AI844951 | 0.001 | -1.23 |  |
| LRRFIP2 | leucine rich repeat (in FLII) interacting protein 2 | AI850587 | 0.007 | -1.20 |  |
| LRRN6A^ | leucine rich repeat neuronal 6A | AI840781 | 0.002 | -1.99 |  |
| **LTC4S** | leukotriene C4 synthase | AI847937 | 0 | -1.19 | * |
| MAL | mal, T-cell differentiation protein | AI839913 | 0.002 | -1.22 |  |
| **MMP14** | matrix metallopeptidase 14 (membrane-inserted) | AI849000 | 0.039 | -1.25 | * |
| **MPHOSPH10** | M-phase phosphoprotein 10 (U3 small nucleolar ribonucleoprotein) | AI844833 | 0.003 | -1.18 |  |
| **MYH11** | myosin, heavy polypeptide 11, smooth muscle | AI846250 | 0.003 | -1.24 |  |
| NDFIP1 | Nedd4 family interacting protein 1 | AI843764 | 0.007 | -1.84 |  |
| NEFL * | neurofilament, light polypeptide 68kDa | AI847934 | 0.008 | -1.27 |  |
| **NF2** | neurofibromin 2 (bilateral acoustic neuroma) | AI842320 | 0 | -1.53 | * |
| **NFKBIA** | nuclear factor of kappa light polypeptide gene enhancer in B-cells inhibitor, alpha | AI845477 | 0.0236 | -1.76 |  |
| **NRXN1** | neurexin 1 | AI853837 | 0.009 | -1.30 |  |
| NRXN2 | neurexin 2 | AI849168 | 0 | -1.44 |  |
| **NSMAF** | neutral sphingomyelinase (N-SMase) activation associated factor | AI849400 | 0.015 | -1.17 |  |
| **OSBP** | oxysterol binding protein | AI854649 | 0.016 | -1.25 |  |
| **P4HA1** | procollagen-proline, 2-oxoglutarate 4-dioxygenase (proline 4-hydroxylase), alpha polypeptide I | AI841136 | 0 | -1.28 |  |
| **PACSIN1** | protein kinase C and casein kinase substrate in neurons 1 | AI840634 | 0.005 | -1.24 |  |
| PCMT1 | protein-L-isoaspartate (D-aspartate) O-methyltransferase | AI853776 | 0.01 | -1.22 | * |
| **PITPNC1** | phosphatidylinositol transfer protein, cytoplasmic 1 | AI854657 | 0.06 | -1.22 | * |
| **PKD1** | polycystic kidney disease 1 (autosomal dominant) | AI852157 | 0.04 | -1.10 |  |
| **PPIL2*** | peptidylprolyl isomerase (cyclophilin)-like 2 | AI837084 | 0.043 | -1.38 |  |
| PPP2R4* | protein phosphatase 2A, regulatory subunit B' (PR 53) | AI851986 | 0.002 | -1.30 |  |
| **PRDX6*** | peroxiredoxin 6 | AI845059 | 0.009 | -1.02 | * |
| PRKAR1A* | protein kinase, cAMP-dependent, regulatory, type I, alpha (tissue specific extinguisher 1) | AI837653 | 0.044 | -1.45 |  |
| **PRKCE** | protein kinase C, epsilon | AI843403 | 0.002 | -1.24 | * |
| PRKCG* | protein kinase C, gamma | AI837227 | 0.004 | -1.34 | * |
| **PTPN1** | protein tyrosine phosphatase, non-receptor type 1 | AI845299 | 0.02 | -1.07 |  |
| **RCBTB2** | regulator of chromosome condensation (RCC1) and BTB (POZ) domain containing protein 2 | AI844477 | 0.001 | -1.19 |  |
| RCL1 | RNA terminal phosphate cyclase-like 1 | AI852608 | 0.001 | -1.24 |  |
| **RGS4*** | regulator of G-protein signalling 4 | AI847765 | 0 | -1.34 |  |
| **RLBP1** | retinaldehyde binding protein 1 | AI849428 | 0.014 | -1.32 |  |
| **RPH3A** | rabphilin 3A homolog (mouse) | AI852149 | 0 | -1.47 |  |
| RRAS | related RAS viral (r-ras) oncogene homolog | AI849261 | 0.005 | -1.40 |  |
| **SCAMP3** | secretory carrier membrane protein 3 | AI847370 | 0.003 | -1.23 |  |
| **SCAMP5** | secretory carrier membrane protein 5 | AI846878 | 0.038 | -1.24 |  |
| **SEPT11** | septin 11 | AI836723 | 0.007 | -1.27 |  |
| **SEPT5*** | septin 5 | AI836045 | 0.044 | -1.26 |  |
| SFRS6 | splicing factor, arginine/serine-rich 6 | AI846595 | 0.013 | -1.10 |  |
| SH3GL1 | SH3-domain GRB2-like 1 | AI853968 | 0 | -2.23 |  |
| **SHC2** | SHC (Src homology 2 domain containing) transforming protein 2 | AI839924 | 0.006 | -1.22 | * |
| **SHMT2** | serine hydroxymethyltransferase 2 (mitochondrial) | AI848189 | 0.03 | -1.20 |  |
| SLC7A10 | solute carrier family 7, (neutral amino acid transporter, y+ system) member 10 | AI837432 | 0.057 | -1.22 |  |
| **SMAD5** | SMAD, mothers against DPP homolog 5 (Drosophila) | AI842842 | 0.016 | -1.23 |  |
| SNPH | syntaphilin | AI835576 | 0.056 | -1.33 |  |
| **SYN2*** | synapsin II | AI843126 | 0.01 | -1.25 | * |
| SYT2* | synaptotagmin II | AI848167 | 0.004 | -1.27 |  |
| **TEF** | thyrotrophic embryonic factor | AI850638 | 0.033 | -1.29 |  |
| **THBS3*** | thrombospondin 3 | AI853483 | 0.003 | -1.33 |  |
| **THY1** | Thy-1 cell surface antigen | AI843835 | 0.008 | -1.19 |  |
| TLN1 | talin 1 | AI849746 | 0 | -1.99 |  |
| **TOB2** | transducer of ERBB2, 2 | AI850962 | 0.002 | -1.20 |  |
| **TUBB2A*** | tubulin, beta 2A | AI849295 | 0.021 | -1.11 | * |
| **VAMP1** | vesicle-associated membrane protein 1 (synaptobrevin 1) | AI850070 | 0.002 | -1.21 | * |
| **VAMP2** | vesicle-associated membrane protein 2 (synaptobrevin 2) | AI848427 | 0.017 | -1.34 | * |
| WBSCR18 | Williams Beuren syndrome chromosome region 18 | AI836921 | 0 | -1.55 |  |
| WSB1 | WD repeat and SOCS box-containing 1 | AI850427 | 0.011 | -1.28 |  |
| **YWHAG*** | tyrosine 3-monooxygenase/tryptophan 5-monooxygenase activation protein, gamma polypeptide | AI848761 | 0.001 | -1.29 |  |
| ZF | HCF-binding transcription factor Zhangfei | AI851171 | 0.006 | -1.20 |  |

Genes in bold type were significantly differentially expressed (FDR <3%) in RML infected mouse brain (n-3) as determined using Agilent whole genome microarrays. Those genes with marked with * have been identified in microarray analysis prion infection in mouse brain in published studies.
