# Supplementary material for: Comprehensive transcriptional profiling of prion infection in mouse models reveals networks of responsive genes
Source: BMC Genomics. 2008 Mar 3;9:114. doi: 10.1186/1471-2164-9-114 (PMC2294129; doi:10.1186/1471-2164-9-114)
Supplement: Additional file 3 — PRG-A list. Candidate PRG genes predominantly expressed in astrocytes or microglia based on profiling experiments performed by Bachoo et al and Ponomarev et al [25,26] [file 1471-2164-9-114-S3.doc]

**Additional File 3, PRG-A list**

| **GENE** | **Fold change** |  |
| --- | --- | --- |
| ABCA1 | 1.46 |  |
| ANXA3 | 1.35 |  |
| APOD | 4.49 |  |
| APOE | 2.21 |  |
| ASRGL1 | 1.33 |  |
| CALM3 | 0.74 |  |
| CEBPD | 1.23 |  |
| CLU | 2.68 |  |
| CST3 | 2.55 |  |
| CTSB | 1.67 |  |
| CTSH | 1.45 |  |
| CTSK | 1.27 |  |
| CTSS | 3.05 |  |
| DHRS1 | 1.95 |  |
| DOCK3 | 0.82 |  |
| FXYD1 | 2.22 |  |
| GFAP | 1.21 |  |
| GJA1 | 1.36 |  |
| GLUD1 | 1.34 |  |
| HRSP12 | 1.41 |  |
| IFIT3 | 1.53 |  |
| ITM2C | 1.30 |  |
| LAMP2 | 1.35 |  |
| LAPTM4A | 1.34 |  |
| LAPTM5 | 1.67 |  |
| LY86 | 2.16 |  |
| MAL | 0.82 |  |
| MBP | 1.48 |  |
| NDFIP1 | 0.54 |  |
| PGCP | 1.24 |  |
| PLP1 | 1.31 |  |
| PRDX6 | 0.98 |  |
| RLBP1 | 0.76 |  |
| S100A1 | 1.37 |  |
| SLC7A10 | 0.82 |  |
| SOX9 | 1.35 |  |
| SPARC | 1.96 |  |
| THY1 | 0.84 |  |
| TOB2 | 0.83 |  |
| VAMP2 | 0.75 |  |
| IIL1A | 4.29 |  |
| TNF | 4.92 |  |
| TGFB1 | 1.35 |  |
| SPP1 | 1.37 |  |
| TF | 2.17 |  |
| B2M | 3.56 |  |
| CRYAB | 1.39 |  |
| HEXB | 2.90 |  |
| RPL10A | 1.44 |  |
| RPL11 | 1.26 |  |
| RPL23 | 1.37 |  |
| RPL24 | 1.48 |  |
| RPL26 | 1.37 |  |
| RPL37A | 1.27 |  |
| RPL4 | 1.28 |  |
| RPL9 | 1.26 |  |
| RPS27 | 1.41 |  |
| RPS3 | 1.30 |  |
| RPS9 | 1.61 |  |
